# Kinetics and characterization of extracellular vesicle release from cells infected with a clinical isolate of respiratory syncytial virus A (ON1 genotype)

**DOI:** 10.1007/s00430-026-00886-y

**Published:** 2026-08-17

**Authors:** Matteo Fracella, Valeria Manganelli, Alessandra Pierangeli, Carolina Scagnolari, Manuel Pacchiele, Eleonora Coratti, Roberta Campagna, Gloria Riitano, Federica Fratini, Agostina Longo, Maurizio Sorice, Guido Antonelli

**Affiliations:** 1 Microbiology and Virology Laboratory, Department of Molecular Medicine, Sapienza University of Rome, Rome, Italy; 2 Department of Experimental Medicine, Sapienza University of Rome, Rome, Italy; 3 Department of Well-being, Health and Environmental Sustainability (BeSSA), Sapienza University of Rome, Rome, Italy; 4 Proteomics Core Facility, Istituto Superiore di Sanità (ISS), Rome, Italy; 5 Sapienza University Hospital “Policlinico Umberto I”, Sapienza University of Rome, Rome, Italy

**Keywords:** Extracellular vesicles, Microvesicles, RSV, Respiratory epithelial cells

## Abstract

Despite the recent approval of preventive measures, the disease burden of respiratory syncytial virus (RSV) infection in children and older adults remains significant. To improve understanding of RSV disease, this study characterized extracellular microvesicles (EMVs) secreted during in vitro infection with a clinical RSV-A (ON1 genotype) strain (RSV/40G-A), isolated in 2022 from a pediatric pneumonia case. The type and content of EMVs released by A549 and BEAS-2B respiratory epithelial cells infected with this clinical isolate were analyzed up to 96 h post-infection (hpi). RSV infection induced a time-dependent increase in EMV levels compared to uninfected controls. EMVs released by infected BEAS-2B and A549 cells contained increasing levels of RSV nucleocapsid protein (RSV-NP) and viral RNA with a peak at 72 hpi. These findings suggest that the kinetics of vesicular release and EMV content are modulated during RSV infection. EMV secretion by host cells may constitute a potential mechanism for intercellular communication or immune activation.

**Supplementary Information:**

The online version contains supplementary material available at 10.1007/s00430-026-00886-y.

## Introduction

Respiratory syncytial virus (RSV) is a ubiquitous, enveloped, negative-stranded RNA virus that belongs to the family *Pneumoviridae*, genus *Orthopneumovirus*. RSV is further differentiated into two antigenic subtypes: A and B [1]. RSV RNA genome is associated with the viral nucleoprotein N in the nucleocapsid, ensuring genome stability and efficient replication within host cells [2]. RSV infection can result in either mild or severe disease depending on several viral and host factors that are not fully understood [3].

 In vitro RSV studies have largely relied on laboratory-adapted strains that have been repeatedly passaged in cell culture [4]. However, serial passaging may introduce adaptive changes that reduce their relevance to currently circulating viruses. Accordingly, clinical-isolate-based RSV strains have shown distinct replication, tropism, and inflammation in vivo compared with laboratory strains [5], as well as substantial phenotypic variability in vitro, including differences in syncytium formation, F protein mRNA levels, progeny production [6], and innate immune responses [7]. These observations support the employment of recent clinical isolates as more physiologically relevant in vitro models.

Within this framework, extracellular vesicles (EVs) have emerged as important mediators of virus–host interactions. Several studies have reported that viruses exploit EVs to transport viral nucleic acids and proteins, leading to significant alterations in the molecular cargo of EVs and modulating the responses of recipient cells [8, 9]. Infections caused by single-stranded RNA viruses, including RSV, have also been shown to involve EV-mediated mechanisms [10–14]; however, the extent to which they contribute to viral replication and pathogenesis is not clearly understood. EVs were first described decades ago, but only in the 1980s were they recognized as bona fide secretory vesicles involved in intercellular communication [15]. Classically, EVs are divided into exosomes, small vesicles released through the multivesicular endosomal pathway, and extracellular microvesicles (EMVs), which are shed from the plasma membrane by outward budding [16]. EVs have been separated and purified in various biological fluids, such as bronchial lavage, breast milk, blood, and saliva [17, 18]. Given their broad functional spectrum and their ability to transfer large biomolecules, EVs represent a promising tool for developing novel therapeutic strategies. In the context of RSV infection, research has mainly focused on exosomes [11–14], whereas the role of EMVs remains less well characterized. Since EMV biogenesis at the plasma membrane shares similarities with the budding of enveloped viruses, EMVs may carry viral RNA and structural proteins. Therefore, understanding their production and release kinetics may provide further insight into RSV infection and pathogenesis. Specifically, in this study, we sought to analyze the kinetics of plasma membrane-derived EMV release from RSV-infected A549 and BEAS-2B cells and to characterize the presence of viral components in this type of EVs, with the aim to clarify their role in cell-to-cell communication. The viral strain used to infect respiratory cells was a clinical isolate of the RSV-A subtype we obtained from a pediatric pneumonia case in October 2022 [19]. This isolate belongs to the ON1 genotype, characterized by an insertion of 72 nucleotides in the gene encoding the outer glycoprotein G, which, since 2012, has replaced the previously circulating RSV-A strains and showed a different innate immune response with respect to its ancestors [20].

Overall, the use of a recent clinical RSV-A isolate allowed us to investigate the kinetics and viral cargo of EMVs in in vitro models that may better preserve biologically relevant virus–host interactions than laboratory-adapted strains.

## Materials and methods

### Clinical RSV strain isolation and sequencing

The clinical RSV isolate, named 40G-A_2022RM (RSV/40G-A), was propagated using ATCC Vero E6 cells, a Vero cell line originally derived from African green monkey kidney epithelial cells. Collection of residual diagnostic specimens from the Virology Laboratory for research studies was approved by the ethics committee of Policlinico Umberto I, Sapienza University Hospital (Prot. 107/12), after the informed consent was obtained from infants’ parent. Briefly, a fresh nasopharyngeal swab specimen that tested positive for RSV using the rapid Xpert^®^ Xpress CoV-2/Flu/RSV plus assay (Cepheid) was selected for viral isolation. To exclude co-infections, a separate aliquot of the same specimen was screened for 14 respiratory viruses (RSV, influenza virus A/B, coronaviruses OC43, 229E, NL-63, HUK1, adenovirus, rhinovirus, parainfluenza 1–3, metapneumovirus, and bocavirus) using reverse transcriptase polymerase chain reactions (RT-PCR), as previously described [19]. Subsequently, a 500 µL aliquot of the unfrozen specimen was used to infect an 80% confluent monolayer of Vero E6 cells in a 12.5 cm^2^ flask. Cells were monitored daily for the onset of cytopathic effects (CPE). At 72 h post-infection (hpi), when CPE was first observed, the cultures were subjected to three freeze–thaw cycles, followed by centrifugation at 2000 rpm for 10 min to remove cellular debris. The clarified supernatant was collected and aliquoted. One aliquot was immediately used to infect fresh Vero E6 cells, whereas the remaining aliquots were stored at − 80 °C. The identity of the resulting clinical RSV isolate, designated RSV/40G-A, was reconfirmed by the RT-PCR assays described above, and the isolate was serially propagated in Vero E6 cells for a total of five passages. The viral titer of the passage-5 stock was determined in 96-well plates of Vero E6 cells using the Reed and Muench method [21] and expressed as the 50% tissue culture infectious dose (TCID₅₀/mL). Sanger sequencing targeting the G gene on the purified viral RNA was performed, as described [22], and the obtained sequences were aligned with that of the 40G-A clinical sample [19] and to ON1 reference sequences, using Bioedit v7.1.3.

### Viral infection and RNA kinetic measurement

ATCC A549 (human lung adenocarcinoma epithelial cell line) and BEAS-2B (a human bronchial epithelial cell line, virally transformed, kind gift from Prof. Nencioni Lucia, Sapienza University, Rome, Italy) were employed to assess viral kinetics and EV production. A549 cells were cultured in Dulbecco’s modified Eagle’s medium (DMEM), while BEAS-2B cells were cultured in RPMI-1640 medium. All media were supplemented with 10% fetal bovine serum (FBS), antibiotic-antimycotic mixture, HEPES buffer, L-glutamine and penicillin/streptomycin solution, and all cell lines were incubated at 37 °C in a humidified atmosphere of 5% CO_2_. Nearly confluent monolayers of A549 and BEAS-2B cells, seeded the day before in 24-well plates with 10% FBS medium depleted of EVs, were infected with RSV/40G-A at a multiplicity of infection (MOI) of 0.01 based on the infectious viral titre determined by TCID_50_ assay on Vero E6 cells using the Reed and Muench method [21]. This low MOI was selected to establish a multicycle infection model suitable for monitoring viral kinetics and EMV release up to 96 hpi while limiting early cytopathic damage and preserving cell integrity for downstream EMV characterization. After 2 h of incubation at 37 °C in a humidified atmosphere of 5% CO_2_, the inoculum was removed, the cells were washed twice with Phosphate-Buffered Saline (PBS), and fresh medium (DMEM for A549 and RPMI-1640 for BEAS-2B) containing 2% FBS (AU-S181T-500), depleted of EVs was added. Specifically, EVdepletion was achieved by a combination of ultracentrifugation (100,000 × g for 12 h at 4 °C) followed by filtration using filters (pore size, 0.22 μm) to remove residual extracellular vesicles. Plates were incubated until 96 hpi at 37 °C and 5% CO_2_. Supernatants were collected from independent wells at each time point (24, 48, 72, and 96 hpi) and directly divided into two aliquots, one for viral RNA detection and the other for EV isolation; the latter was processed immediately for EMV purification, as described in the subsequent Methods section. In parallel, the corresponding cell monolayers were lysed in TRIzol reagent and collected by scraping to ensure complete recovery of the lysate. The same conditions were applied to uninfected control cells. TRIzol lysates and supernatant aliquots for viral RNA analysis were stored separately at − 80 °C until further processing.

Total RNA was extracted from cell lysates and supernatants using the Direct-zol RNA Miniprep Plus Kit (Zymo Research, Irvine, CA) according to the manufacturer’s instructions, including on-column DNase I treatment. To minimize technical variability, equal starting volumes were used within each fraction, and all samples were processed in parallel using the same extraction kit and procedures. Briefly, RNA concentration and purity were assessed by measuring absorbance at 260 and 280 nm using a NanoDrop Microvolume Spectrophotometer (Thermo Fisher Scientific, Waltham, MA). A total of 500 ng of RNA was reverse transcribed using the High-Capacity cDNA Reverse Transcription Kit (Applied Biosystems, Foster City, CA), according to the manufacturer’s instructions. The absolute number of RSV-nucleocapsid (N) gene copies was quantified at each time point (24, 48, 72, and 96 hpi) by digital PCR (dPCR) (QIAcuity Probe PCR Kit; QIAGEN, Hilden, Germany), employing the same TaqMan primers and probe set described by Pierangeli et al. [22]. Specifically, forward and reverse primers (8 µM), probe (4 µM) and 5 µL template cDNA were added to the Probe PCR Master Mix (QIAcuity Probe PCR Kit) in a 40 µL reaction volume. The reaction was run on the QIAcuity One 5plex instrument (QIAGEN, Hilden, Germany), with 26,000 partitions generated by the 24-well 26K nanoplate. TaqMan-based dPCR was performed under the following conditions: 95 °C for 2 min, followed by 40 cycles of 95 °C for 15 s and 60 °C for 30 s. Finally, all wells were imaged (green channel) with an exposure time of 500 ms. No-template controls (NTCs) were included in each run for quality control. Data were analyzed using QIAcuity Software Suite v2.5.0.1, and results were expressed as absolute copies/µL after Poisson correction. Accordingly, RSV-N quantification was standardized according to an equivalent total-RNA input.

### Extracellular microvesicle purification from cultured cells

BEAS-2B and A549 cell supernatants collected from both RSV/40G-A-infected and uninfected wells at different time points (24, 48, 72, and 96 hpi) were first centrifuged at 3,000 × g for 5 min to remove debris. The recovered supernatants were then transferred to a new tube and subjected to two consecutive centrifugations at 15,000 × g for 30 min to isolate EMVs, with the second centrifugation performed after resuspension in filtered PBS (0.22 μm). The resulting pellets were divided into three aliquots: one for NanoSight analysis, one for western blot analysis and the last one for viral RNA quantification. Viral RNA quantification in the EMVs purified at the different time points was performed by dPCR, as described above for the intracellular and extracellular fractions, under the same experimental and analytical conditions for A549 and BEAS-2B.

### NanoSight analysis

The concentration and the size distribution of EMVs from BEAS-2B and A549 cell supernatants collected from both RSV/40G-A-infected and uninfected wells were measured by nanoparticle tracking analysis (NTA). EMV pellets were resuspended in PBS, and 20 µL were used for NTA analysis by a NS300 instrument (Malvern, UK, Software Version 3.4). To get a suitable concentration, the samples were diluted in freshly filtered PBS 2.5 × 10^2^ and vortexed for 1 min. Samples were flowed at a syringe pump speed of 30 AU. NTA was used to determine particle concentration and size distribution, whereas vesicle identity was assessed by immunoblotting and flow-cytometric marker analysis (Supplementary Fig. 2), as detailed below.

### Characterization of EMVs by immunoblotting and flow cytometric analyses

EMVs from BEAS-2B and A549 cell supernatants collected from both RSV/40G-A-infected and uninfected wells were lysed in RIPA buffer and subjected to SDS-PAGE. Specifically, protein concentration was determined using the Bio-Rad protein assay (Bio-Rad) and 20 µg of total protein were loaded per lane. The proteins were then transferred onto PVDF membranes (Bio-Rad, 162–0177). To prevent nonspecific binding, the membranes were blocked with a solution containing 1% bovine serum albumin (BSA) in Tris-buffered saline (TBS) (Bio-Rad, 1706435) with 0.05% Tween 20 (Bio-Rad, 1706531). The membranes were probed with the following antibodies: mouse anti-RSV nucleocapsid protein (RSV-NP) (Sigma Aldrich, MAB858-3, 1:500, 42–44 kDa), mouse anti-Annexin A1 (ANXA1) (Santa Cruz Biotechnology, 12740, 1:500, 37 kDa) and rabbit anti-CD81 (Abcam, ab155760, dilution 1:500, 25 kDa). The interaction was visualized using horseradish peroxidase (HRP)-conjugated secondary antibodies [anti-rabbit (A1949-1VL, 1:1000) and anti-mouse (NA931V, Sigma-Aldrich, 1:1000)]. Immunoreactivity was detected through a chemiluminescence reaction, using the ECL western detection system (Amersham, RPN2106). Of note, for CD81 detection, fractions were subjected to SDS-PAGE under nonreducing conditions [23]. Densitometric scanning analysis was performed using NIH Image J 1.62 software. To further assess ANXA1 expression, EMVs isolated from uninfected and RSV/40G-A-infected BEAS-2B cell supernatants were stained with anti-ANXA1 antibody (Santa Cruz Biotechnology, 12740), followed by a FITC-conjugated secondary antibody (Sigma Aldrich, F5262). Autofluorescence and isotype controls were included. Samples were analyzed using a CytoFLEX flow cytometer (Beckman Coulter).

### Statistical analysis

Data of viral RNA copies, in the intracellular and extracellular compartments and in the purified EMVs are presented as means ± standard deviation (SD) of three independent experiments. Student’s t-test was used to assess statistical differences in EMV concentrations (expressed as particles/mL) and in western blot densitometric levels of RSV-NP protein normalized to ANXA1 protein between uninfected and RSV/40G-A-infected BEAS-2B and A549 cells. Differences in RSV RNA copies over time were evaluated using one-way analysis of variance (ANOVA), followed by post hoc multiple comparison tests with Bonferroni correction. Comparisons of viral RNA levels between the two cell lines (BEAS-2B and A549) at each time point (24, 48, 72, and 96 hpi) in the intracellular and extracellular compartments, as well as in the purified EMVs, were performed using a Student’s t-test.

## Results

### Mutational analysis of the ON1 clinical isolate G gene

A residual sample from a nasopharyngeal swab of a two-year-old child with a clinical diagnosis of viral pneumonia that tested positive for RSV was obtained during a previous study [19] and used for viral isolation on Vero E6 cells, as described in the Methods section. The resulting clinical isolate was designated RSV/40G-A. To ensure that the cell culturing process did not introduce relevant mutations, the G gene of the RSV/40G-A isolate was sequenced at passages one and five. The sequences obtained at both passages were identical to that of the original clinical specimen [19]. Hence, the ON1 strains from the clinical sample and the viral isolate both belonged to RSV-A clade A.D.3, which was dominant in the post-pandemic period [19]. The ON1 strains were characterized by 19 amino acid substitutions in the G protein compared to the NextStrain reference sequence, hRSV/A/England/397/2017 (EPI_ISL_412866).

### Kinetics and size of EMVs released during RSV/40G-A infection by NTA analysis

**Fig. 1:**
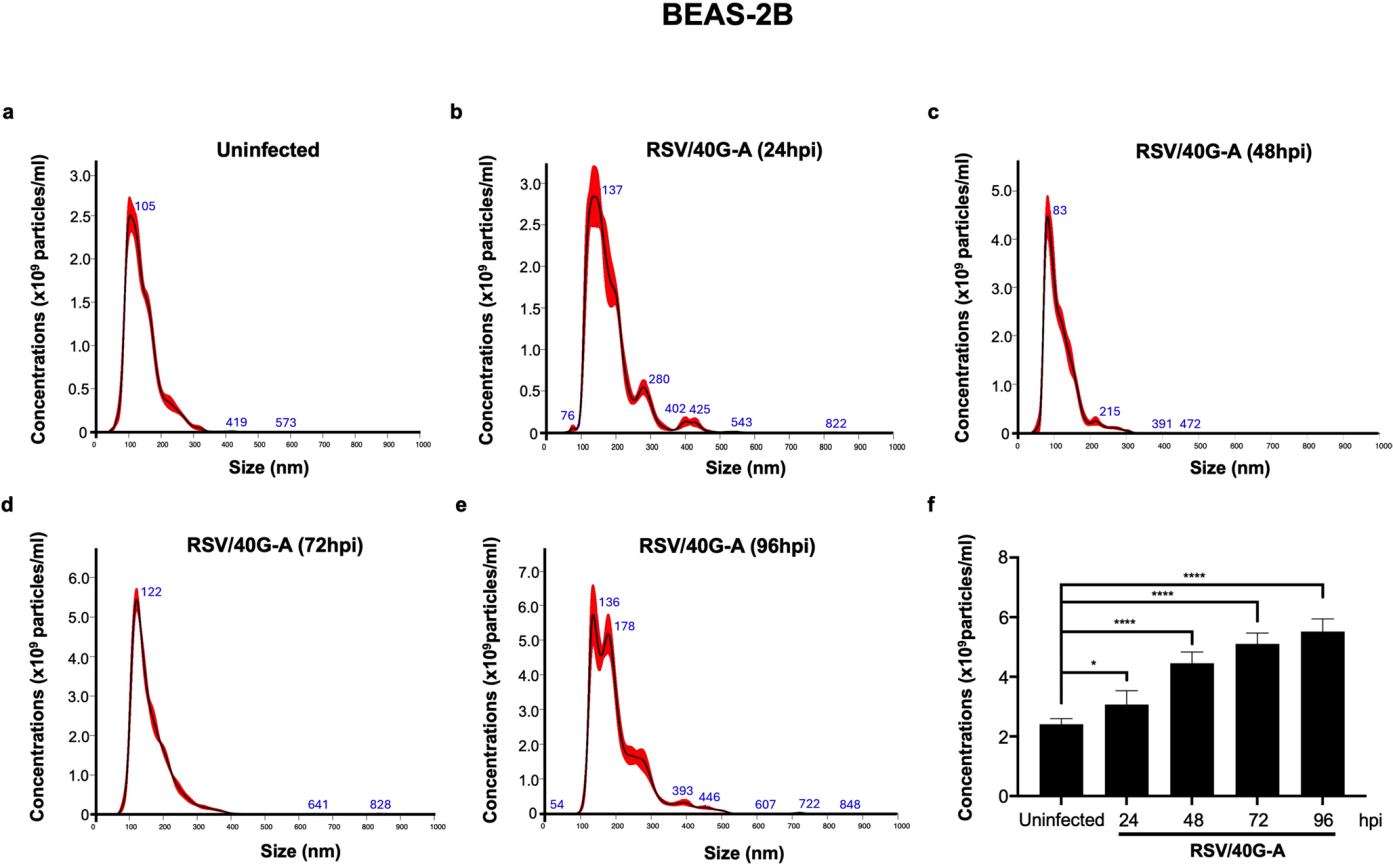
Concentration and size of EVs released by BEAS-2B cells measured by nanoparticle tracking analysis (NTA). Nanoparticle tracking analysis (NTA) quantification of extracellular microvesicles (EMVs) (Panels **a**–**e**), expressed as particles/mL, was performed on supernatants from BEAS-2B cells, both uninfected and infected with RSV/40G-A, at different time points (24, 48, 72, and 96 hpi). NTA software allows the analysis of video images of the particle movement calculating the mean size (blue number) and particle concentration values. The curve describes the relationship between particle number distribution (left Y-axis) and particle size (X-axis). Panel **f** shows the quantitative comparison of EMV concentration in uninfected and RSV/40G-A conditions. For statistical evaluation, the values are expressed as mean ± SD of three independent experiments, performed to minimize experimental variability, and compared using the Student’s t-test. **** *p* < .0001

Since EVs play a key role in cell-to-cell communication and are increasingly recognized as modulators of viral infection, this study aimed to establish whether RSV infection impacts EMV release. Therefore, BEAS-2B and A549 monolayers were infected with the clinical isolate RSV/40G-A at an MOI of 0.01 and monitored up to 96 hpi under multicycle infection conditions in three independent experiments. Syncytia formation first appeared at 72 hpi and increased at 96 hpi, when cell detachment was observed. Infectious virion production was supported by serial passage of culture supernatants in the same cell lines, as assessed by cytopathic effect/syncytia formation and TCID_50_ titration (data not shown). As shown in Fig. 1, the analysis of EMVs derived from BEAS-2B cell supernatants at 24, 48, 72, and 96 hpi was performed using NTA. As expected, the mean size of EMVs was not significantly modified at the different time points. Notably, RSV infection elicited a time-dependent increase in EMV concentration, rising from an average of 3 × 10^9^ particles/mL at 24 hpi to 4.4 × 10^9^ particles/mL at 48 hpi, 5 × 10^9^ particles/mL at 72 hpi, and 5.5 × 10^9^ particles/mL at 96 hpi, compared to uninfected cells (2.4 × 10^9^ particles/mL), increasing to approximately 1.25-fold at 24 hpi, 1.83-fold at 48 hpi, 2.08-fold at 72 hpi, and 2.29-fold at 96 hpi compared to uninfected cells (Fig. 1). Similar results were obtained in A549 cells (Supplementary Fig. 1). These findings show a time-dependent increase in particles within the EMV-enriched fraction during RSV infection. The vesicular nature of this fraction was supported by marker-based characterization, including immunoblotting and flow cytometry, as described below, whereas NTA was used to assess particle concentration and size distribution. Moreover, no major shifts in particle size distribution were observed over time, suggesting that RSV infection was mainly associated with an increase in particle abundance within the EMV-enriched fraction rather than with selective enrichment of specific EMV subtypes.

### Characterization of viral components within EMVs produced during RSV/40G-A infection

To assess the presence of viral RNA, we analyzed the content of EMVs isolated from RSV/40G-A-infected BEAS-2B and A549 cells and measured viral replication kinetics. Purified viral RNA was quantified by dPCR in EMVs, as well as in the intracellular and extracellular fractions of both cell models, at 24, 48, 72 and 96 hpi. For EMV purification, supernatants from BEAS-2B and A549 cell cultures, both RSV/40G-A-infected and uninfected, were subjected to sequential centrifugation, as described in the Methods section.

**Fig. 2:**
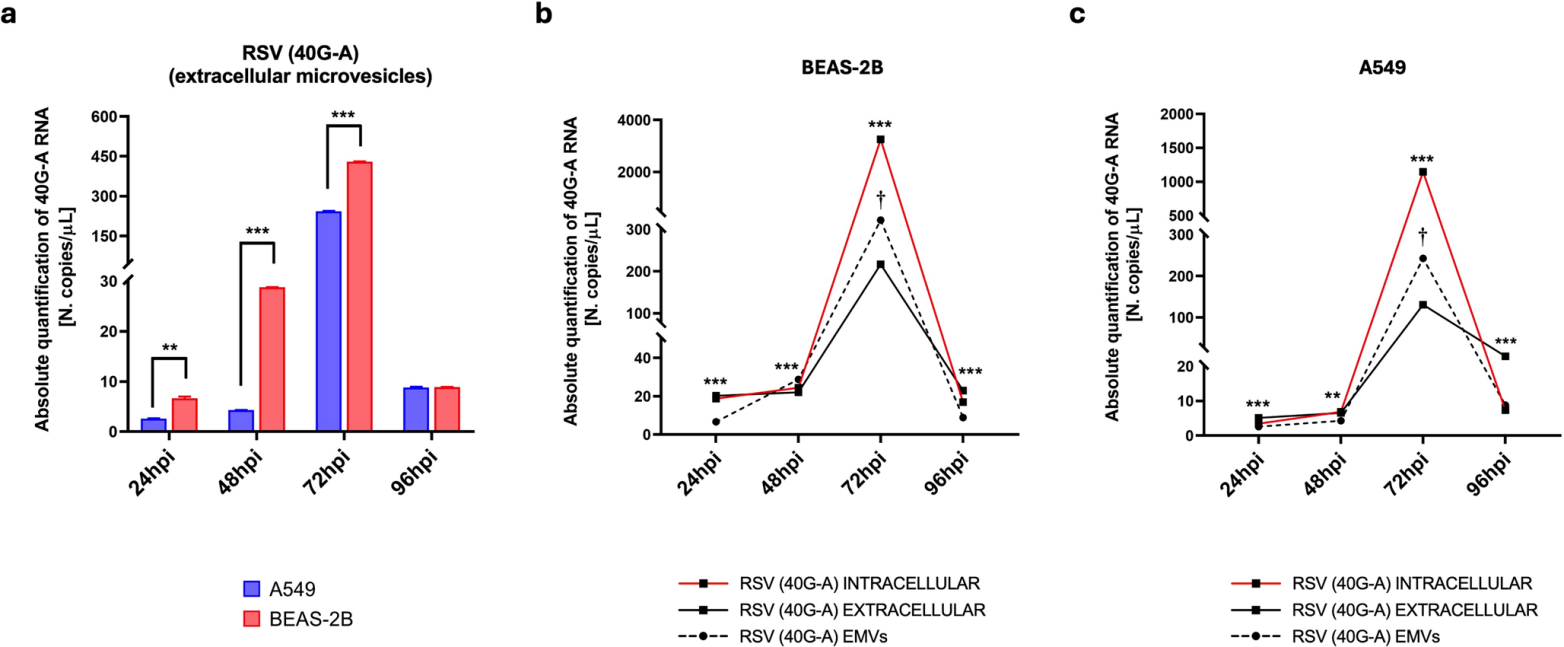
Viral RNA content and kinetic in the EMVs released by RSV/40G-A-infected cell lines. Panel **a:** Comparison of RSV/40G-A-RNA quantification in the extracellular microvesicles (EMVs) purified from infected BEAS-2B and A549 at the different time points. Panels **b** and **c**: RSV/40G-A-RNA quantification in the purified EMVs and in the intracellular and extracellular comparts of the infected BEAS-2B and A549 cells. Absolute quantification of RSV/40G-A RNA (N. copies/µl) was performed at 24, 48, 72, and 96 hpi, targeting the RSV nucleocapsid (N) gene, in the purified EMVs released by BEAS-2B and A549 cells (Panel **a**) and in the intracellular and extracellular comparts of BEAS-2B (Panel **b**) and A549 cells (Panel **c**). Data are presented as mean ± standard deviation (SD) of three independent experiments. RSV RNA copies over time in EMVs isolated from BEAS-2B and A549 cells were compared using one-way analysis of variance (ANOVA) followed by post hoc multiple comparison tests with Bonferroni correction [72 hpi vs. 24 hpi, 48 hpi, 96 hpi (†; *p *< .001)]. Comparisons of viral RNA levels in EMVs between the two cell lines at each time point were conducted using the Student’s t-test (Panel **a**). Comparisons of viral RNA levels among the intracellular, extracellular compartments, and in the purified EMVs at each time point were performed using one-way ANOVA in BEAS-2B (Panel **b**) and A549 (Panel **c**) cell models. **p < .01; ***p < .001

RSV/40G-A RNA levels (N. copies/µl) peaked at 72 hpi in the purified EMVs of both BEAS-2B (429.76 ± 0.50) and A549 (242.70 ± 1.97) cells (*p* < .001 for RNA levels between 72 hpi vs. 24 hpi, 48 hpi and 96 hpi), as shown in Fig. 2, panel a. Overall, EMVs released by BEAS-2B cells contained a higher viral RNA copies over time compared to A549 cells (*p* = .003 for 24 hpi, *p* < .001 for 48hpi and *p* < .001 for 72hpi) (Fig. 2, panel a).

Consistent with the viral RNA content of EMVs, the highest amounts of RSV/40G-A RNA (N. copies/µl) were detected at 72 hpi in both the intracellular and extracellular compartments with a sharp decrease at 96 hpi (Fig. 2, panels b and c). The viral RNA levels in EMVs were subsequently compared with those measured in the intracellular and extracellular fractions. Notably, viral RNA copies were significantly higher in the intracellular fraction of both cell lines (3254.66 ± 1.35 for BEAS-2B and 1146.70 ± 0.69 for A549), followed by intermediate levels in EMVs (429.76 ± 0.50 for BEAS-2B and 242.70 ± 1.97 for A549), with the lowest amount found in the extracellular fraction (216.95 ± 0.64 for BEAS-2B and 130.65 ± 1.06 for A549) (*p* < .001 for RNA levels at 72 hpi between EMVs, intracellular and extracellular compartments; Fig. 2, panels b and c; Supplementary Table 1). The coincident peak observed at 72 hpi across compartments likely reflects the time of maximal overall viral burden in this multicycle infection model, rather than a strict temporal separation between intracellular replication and extracellular release.

**Fig. 3:**
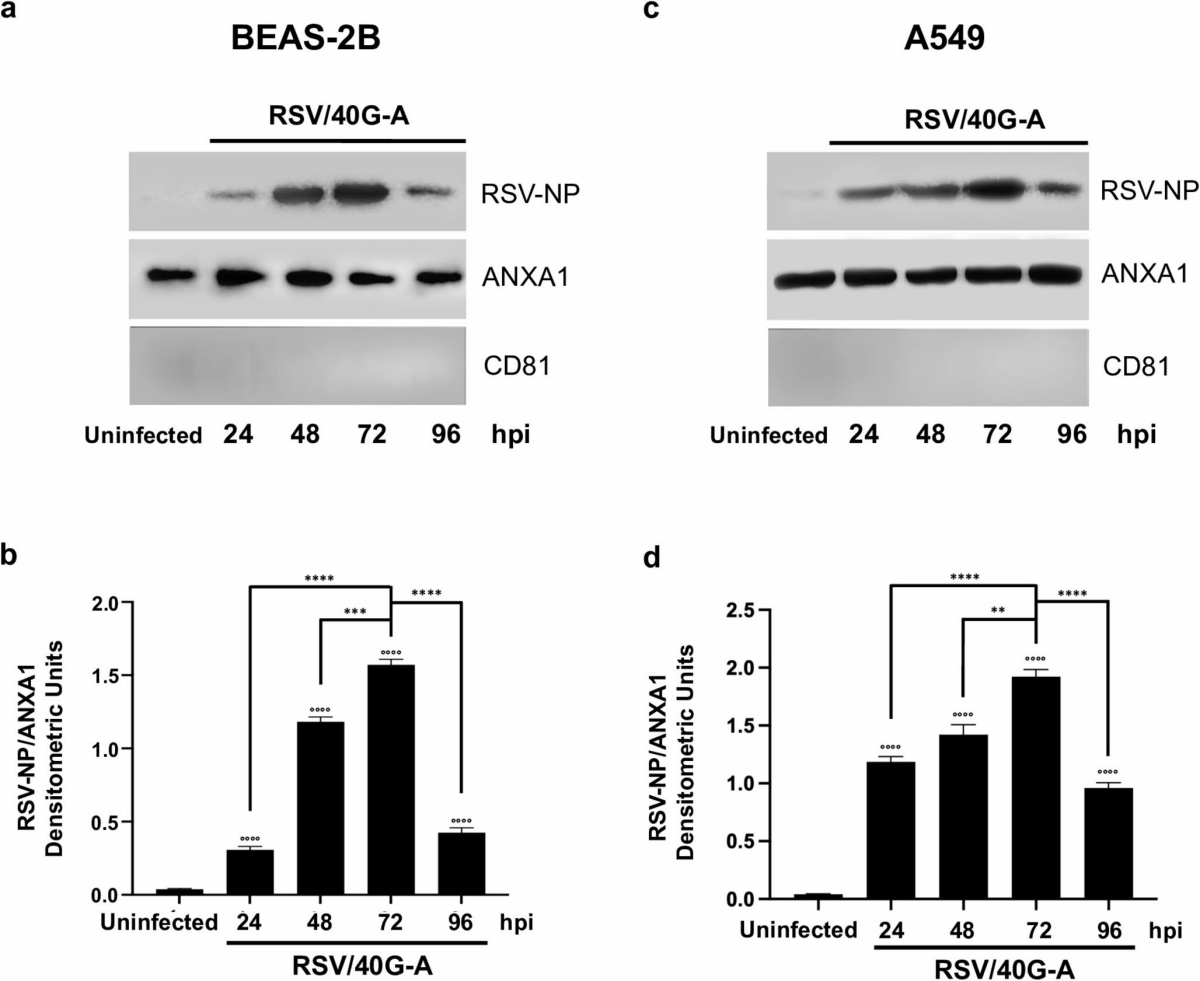
Characterization of EMVs released by BEAS-2B and A549 cells after RSV/40G-A infection. In the same RSV/40G-A infection experiments, as described in Methods, immunoblots of extracellular microvesicles (EMVs) derived from BEAS-2B (Panel **a**) and A549 (panel **c**) cells, either uninfected or infected with RSV/40G-A (24, 48, 72 and 96 hpi), were performed using anti-RSV nucleoprotein (RSV-NP), anti-Annexin A1 (ANXA1) and anti-CD81 antibodies. The blots shown represent one of three independent experiments with similar results. Bar graphs in Panels **b** (BEAS-2B) and **d** (A549) show densitometric analysis of RSV-NP levels normalized to ANXA1. Data are presented as mean ± SD from three independent experiments. Student’s t-test was used to assess statistical differences in densitometric values of RSV-NP between uninfected and RSV/40G-A-infected cell models (BEAS-2B and A549; °°°° *p* < .0001). Additional comparisons were performed between 72 hpi and the other experimental time points (72 vs. 24, 48, and 96 hpi; ** *p* < .01; *** *p* < .001; **** *p* < .0001).

To further characterize the presence of viral components within EMVs, immunoblotting analysis was performed in the same RSV infection experiments (Fig. 3), as described in Methods. The RSV-NP was enriched in EMVs released from RSV/40G-A-infected cells at 24, 48, 72, and 96 hpi, compared to the control (*p* < .0001). Notably, densitometric analysis showed significantly higher levels of RSV-NP at 72 hpi in both cell lines compared with 24, 48, and 96 hpi (BEAS-2B: *p* < .0001, *p* = .0002, and *p* < .0001, respectively; A549: *p* < .0001, *p* = .0012, and *p* < .0001, respectively) (Fig. 3, panels b and d).

As expected, Annexin A1 (ANXA1), a specific marker for plasma membrane-derived EVs, was also evenly detected at all time points, whereas CD81, a specific exosome marker, was virtually absent in the EMV samples (Fig. 3). Flow cytometric analysis further confirmed ANXA1 expression in EMVs derived from both uninfected and RSV/40G-A-infected BEAS-2B cells (Supplementary Fig. 2).

## Discussion

Characterization of RSV-host cell interactions has been the subject of numerous studies, but few have addressed the important issue of the kinetics of EV production and composition following RSV infection [11–14]. Furthermore, these studies used laboratory strains of RSV that were adapted long ago for growth in cell culture and are therefore not representative of contemporary strains.

Hence, we investigated the kinetics of EMV production and composition following infection with a clinical RSV-A strain of the ON1 genotype isolated in 2022 (RSV/40G-A), employing respiratory epithelial cell lines. Specifically, we used the airway epithelial cells BEAS-2B and A549, which are widely established as in vitro models of RSV infection [4, 24]. Despite originating from different tissues - the bronchial and alveolar epithelium, respectively - the two cell models exhibited similar kinetics of EMV production (Fig. 1 and Supplementary Fig. 1) and RSV-induced cytopathic effects. In particular, NTA analysis revealed a progressive increase in EMV concentration following RSV/40G-A infection, consistent with a time-dependent increase in the shedding of plasma membrane-derived vesicles. Marker-based characterization by immunoblotting and flow cytometry further supported the vesicular nature of the isolated fraction (Fig. 3 and Supplementary Fig. 2). In this context, EMVs may not only represent promising biomarkers of RSV infection, but also play an active and pivotal role in shaping disease pathogenesis.

Viral RNA quantification and immunoblotting detected RSV-N RNA and RSV-NP protein in EMVs over time, with the highest levels at 72 hpi (Figs. 2 and 3), consistent with the peak viral burden in the cultures. The higher RSV RNA content observed in BEAS-2B-derived EMVs than in A549-derived EMVs may reflect cell-specific responses to RSV infection. Previous studies have shown that BEAS-2B cells display a stronger intrinsic antiviral profile than A549 cells [24], which may influence viral RNA processing and incorporation into EMVs [12, 14].

The associations of viral components with EVs have been reported in RSV [12] and in other RNA virus infections, including Hepatitis C [25, 26], Hepatitis A [27], Zika [28] and Chikungunya [29]. However, most available studies have mainly focused on exosomes rather than on plasma membrane-derived microvesicles/EMVs and their production kinetics. In this regard, our findings extend the current literature by showing a time-dependent accumulation of viral material within EMVs during infection. Notably, EMVs were found to contain higher amounts of viral RNA compared to the corresponding supernatant at 72 hpi (Fig. 2 and Supplementary Table 1), which likely reflects viral RNA packaging rather than a passive release of virions. This enrichment may also result from protection of viral RNA from extracellular degradation, differential release efficiencies between virions and vesicles, or host antiviral responses limiting virion production at later stages of infection. By contrast, the marked decline in viral RNA copies and protein levels observed across all compartments at 96 hpi (Figs. 2 and 3) may result from reduced cell viability with a consequent impairment in the synthesis of viral components, suggesting that EMV production at late stages of infection predominantly reflects cellular stress or death induced by the infection.

The detection of ANXA1, a marker of plasma membrane-derived EVs [30], together with the absence of CD81, a canonical tetraspanin protein ubiquitously expressed on exosomes and commonly used as a definitive exosomal marker [31], supports the enrichment of the isolated fraction in plasma membrane-derived EMVs rather than exosomes (Fig. 3 and Supplementary Fig. 2). Unlike exosomes, which originate from the endosomal pathway, EMVs are generated by outward budding of the plasma membrane [16, 23]. This distinction may be particularly relevant to RSV infection, as viral assembly and egress are also closely associated with the plasma membrane and its recycling machinery [32, 33]. The spatial proximity of these processes may facilitate the incorporation of viral RNA and proteins into EMVs. Depending on their cargo and recipient cells, RSV-associated EMVs may exert either proviral or antiviral effects, as previously reported for RSV-induced EVs [12, 14]. Accordingly, the presence of RSV nucleoprotein within EMVs (Fig. 3) suggests that these vesicles may contribute to the transfer of viral components to neighboring cells, including cells not directly reached by infectious virions. Overall, it is possible to hypothesize that the enhanced release of EMVs following RSV infection may reflect a host defense mechanism, a viral strategy to modulate the immune response, or a mechanism facilitating viral spread through the EMV-mediated transfer of viral components.

In conclusion, our study highlights the dynamic interplay between RSV infection and EMV release, providing new insights into the mechanisms of virus-host interactions. Further characterization of EMV-associated viral components is needed to determine whether they are able to activate an antiviral response in uninfected cells, and whether they may play a role in transmission to recipient cells. Although the experimental workflow was standardized using the same processing conditions and equal total-RNA input, dedicated process controls could further refine comparisons among cellular, extracellular, and EMV-enriched fractions and should be incorporated into subsequent investigations. Moreover, direct comparison with a laboratory-adapted RSV-A strain may provide additional insight and warrants investigation in future studies. Overall, understanding the role of RSV-induced EMVs in infection progression may open new avenues for therapeutic strategies targeting EMV-mediated viral dissemination and immune modulation.

## Supplementary Information

Below is the link to the electronic supplementary material.

Supplementary Material 1.

## Data Availability

The datasets generated and analyzed during the current study are available from the corresponding author upon reasonable request.
