# Supplementary material for: Kinetics and characterization of extracellular vesicle release from cells infected with a clinical isolate of respiratory syncytial virus A (ON1 genotype)
Source: Med Microbiol Immunol. 2026 Aug 17;215(1):23. doi: 10.1007/s00430-026-00886-y (PMC13481564; doi:10.1007/s00430-026-00886-y)
Supplement: Supplementary file 1 [file 430_2026_886_MOESM1_ESM.pdf]

MEDICAL MICROBIOLOGY AND IMMUNOLOGY

Supplementary information for **Kinetics and characterization of extracellular vesicle release from cells infected with a clinical isolate of respiratory syncytial virus A (ON1 genotype)**

Matteo Fracella[1✉^], Valeria Manganelli[2^], Alessandra Pierangeli[3], Carolina Scagnolari[1], Manuel Pacchiele[1], Eleonora Coratti[1], Roberta Campagna[1], Gloria Riitano[2], Federica Fratini[4], Agostina Longo[2], Maurizio Sorice[2,5°], Guido Antonelli[1,5°].

1. Microbiology and Virology Laboratory, Department of Molecular Medicine, Sapienza University of Rome, Rome, Italy;
2. Department of Experimental Medicine, Sapienza University of Rome, Rome, Italy;
3. Department of Well-being, Health and Environmental Sustainability (BeSSA), Sapienza University of Rome, Rome, Italy;
4. Proteomics Core Facility, Istituto Superiore di Sanità (ISS), Rome, Italy;
5. Sapienza University Hospital "Policlinico Umberto I", Sapienza University of Rome, Rome, Italy.

^These authors contributed equally to this work

° These authors contributed equally and jointly supervised this work.

✉ CORRESPONDING AUTHOR:

Matteo Fracella (e-mail: matteo.fracella@uniroma1.it)

Microbiology and Virology Laboratory, Department of Molecular Medicine, Sapienza University of Rome, Rome, Italy.

ACKNOWLEDGMENTS

This work was supported by European Union funding within the NextGeneration EU-MUR PNRR Extended Partnership initiative on Emerging Infectious Diseases (Project n. PE00000007, INF-ACT).

AUTHOR CONTRIBUTIONS

Matteo Fracella: investigation, data curation, methodology, writing - original draft; **Valeria Manganelli**: investigation, data curation, methodology, writing - original draft; **Alessandra Pierangeli**: conceptualization, supervision, validation, writing - review & editing; **Carolina Scagnolari**: validation, writing - review & editing; **Manuel Pacchiele**: investigation; **Eleonora Coratti**: investigation; Roberta Campagna: investigation; **Gloria Riitano**: investigation, writing - review & editing; **Federica Fratini**: investigation; **Agostina Longo**: supervision, writing - review & editing; **Maurizio Sorice**: project administration, conceptualization, supervision, writing - review & editing; **Guido Antonelli**: project administration, conceptualization, supervision, funding acquisition, writing - review & editing.

Supplementary Table 1. RSV/40G-A RNA kinetics in BEAS-2B and A549 cell models

Cell model	Time points	RSV/40G-A RNA copies (n. copies/µl)			*P-value (A vs. B vs. C)*	*P-value (A vs. B)*	*P-value (A vs. C)*	*P-value (B vs. C)*
		Intracellular (A)	Extracellular (B)	Extracellular MV (C)				
BEAS-2B	24hpi (mean ± SD)	18.79 ± 0.16	20.24 ± 0.06	6.65 ± 0.33	<.001	.019	<.001	<.001
	48hpi (mean ± SD)	24.47 ± 0.20	22.04 ± 0.10	28.79 ± 0.03	<.001	<.001	<.001	<.001
	72hpi (mean ± SD)	3254.66 ± 1.35	216.95 ± 0.64	429.76 ± 0.50	<.001	<.001	<.001	<.001
	96hpi (mean ± SD)	16.92 ± 0.28	22.96 ± 0.37	8.85 ± 0.08	<.001	<.001	<.001	<.001
A549	24hpi (mean ± SD)	3.47 ± 0.17	5.14 ± 0.05	2.62 ± 0.06	<.001	.002	.013	<.001
	48hpi (mean ± SD)	6.87 ± 0.33	6.57 ± 0.06	4.29 ± 0.03	.002	.642	.003	.004
	72hpi (mean ± SD)	1146.70 ± 2.69	130.65 ± 1.06	242.70 ± 1.97	<.001	<.001	<.001	<.001
	96hpi (mean ± SD)	7.36 ± 0.11	21.40 ± 0.20	8.81 ± 0.13	<.001	<.001	.007	<.001

RSV/40G-A RNA kinetics were assessed by digital PCR (dPCR) using a TaqMan probe targeting the RSV nucleocapsid (N) gene. Absolute quantification of viral RNA (copies/µl) was performed at 24-, 48-, 72-, and 96-hours post-infection (hpi) in the extracellular microvesicles (EMVs), intracellular and extracellular-associated compartments of BEAS-2B and A549 cells. Data are presented as mean ± standard deviation (SD). Differences in RSV RNA levels (copies/µl) among the intracellular, extracellular, and EMV-associated compartments in BEAS-2B and A549 cells at each time point were evaluated by one-way ANOVA (A vs B vs C), followed by post hoc multiple comparison tests with Bonferroni correction (pairwise comparisons: A vs B; A vs C; B vs C).

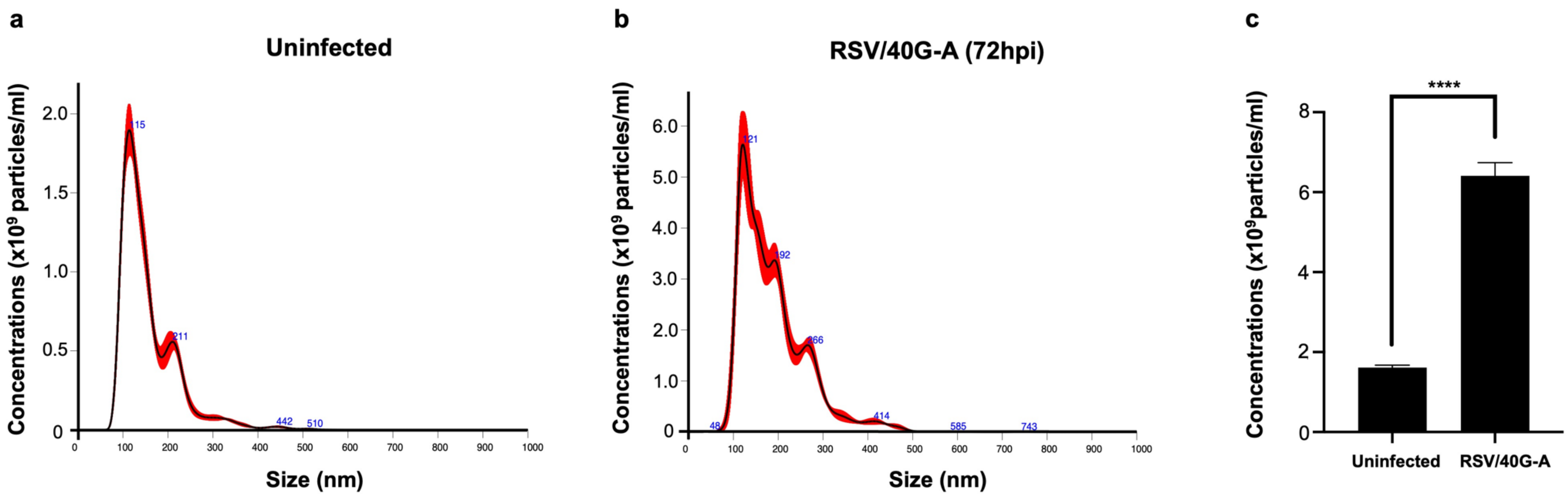

Supplementary Fig.1 Concentration and size of EMVs released by A549 cells measured by nanoparticle tracking analysis (NTA)
NTA quantification of extracellular microvesicles (EMVs) (Panels a-b), expressed as particles/mL, was performed on supernatants from A549 cells, both uninfected and infected with RSV/40G-A, at 72 hours post infection (hpi). For statistical evaluation, the values are expressed as mean ± SD of three independent experiments and compared using the Student's t-test (Panel c). **** $p < .0001$.

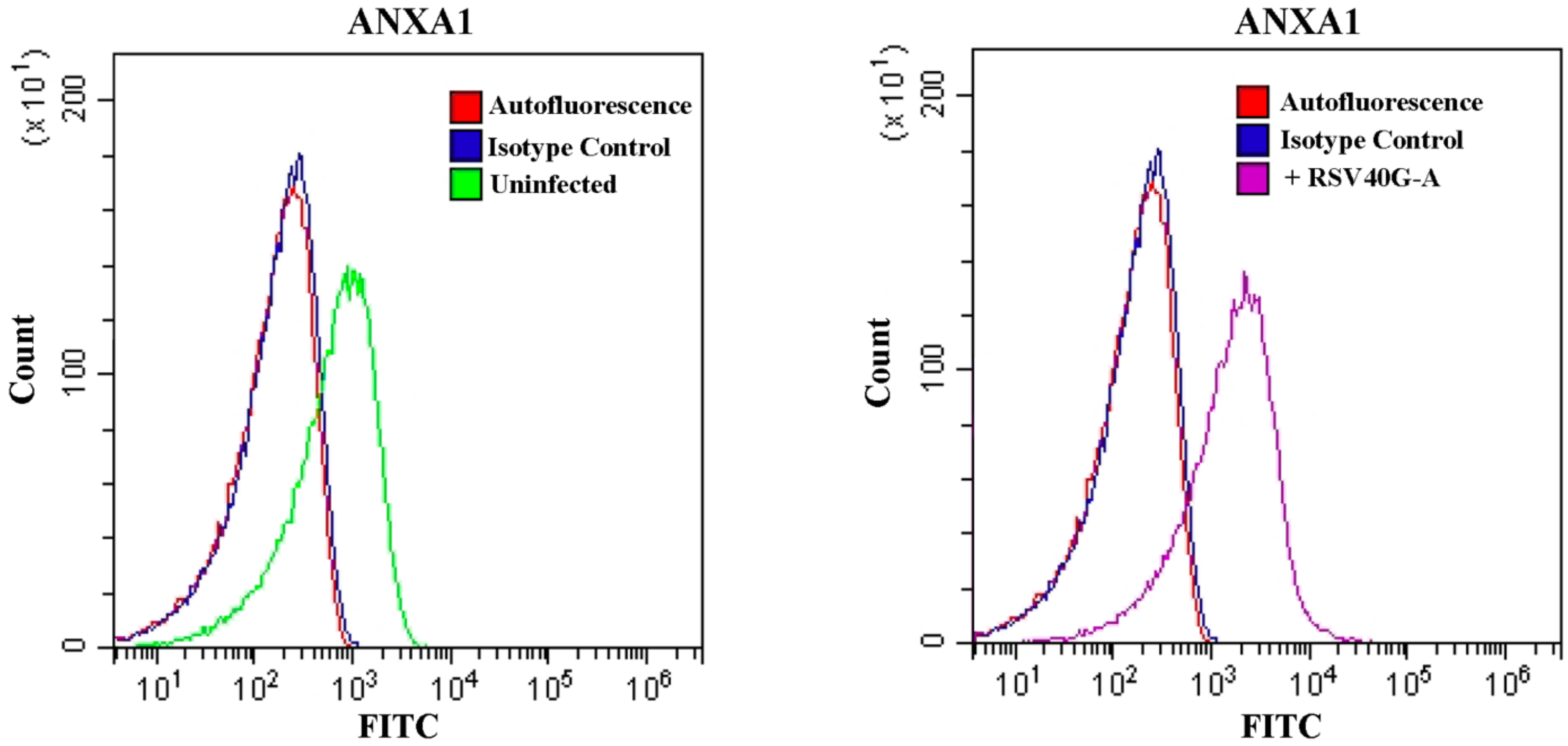

Supplementary Fig.2 ANXA1 expression on EMVs by flow cytometric analysis

Extracellular microvesicles (EMVs) obtained from BEAS-2B, uninfected or infected with RSV/40G-A, were stained with anti-Annexin A1 (ANXA1) antibody, followed by FITC-conjugated secondary antibodies. Flow cytometry was performed using a CytoFLEX cytometer (Beckman Coulter).
